# Surgical treatment of myasthenia gravis: 10 years of single center experience

**DOI:** 10.3389/fneur.2025.1595927

**Published:** 2025-08-07

**Authors:** José De Sá Moraes Neto, Jaqueline Schaparini Fonini, Francisco De Assis Cavalcanti Neto, Fernanda Aquino De Oliveira, Gabriel Lunardi Aranha, Antonio Alberto Zambon, Eduardo De Paula Estephan, Edmar Zanoteli, Paulo Manuel Pêgo-Fernandes, Alessandro Wasum Mariani

**Affiliations:** ^1^University of São Paulo, São Paulo, Brazil; ^2^Instituto do Coracao, Hospital das Clinicas HCFMUSP, Faculdade de Medicina, Universidade de São Paulo, São Paulo, Brazil; ^3^Hospital das Clínicas, São Paulo, Brazil

**Keywords:** myasthenia, tymoma, thymectomy, Osserman classification, minimally invasive thoracic surgery

## Abstract

**Objective:**

To evaluate a 10-year experience in the surgical treatment of patients with myasthenia gravis, focusing on clinical and pharmacological outcomes.

**Methods:**

A retrospective single-center study was conducted based on prospective data collection from the Redcap® maintained by the Thoracic Surgery Department in a Brazilian tertiary hospital. Patients with myasthenia gravis (AchR positive), who underwent surgical treatment as an adjuvant to clinical therapy were included.

**Results:**

The study comprised 85 patients with a mean age of 43 years; 75% were female. Regarding the type of surgery, 24.7% underwent sternotomy and 75.3% minimally invasive approach. The median hospital stay was 4.0 days (p25 = 3.0; p75 = 5.5), and the median ICU stay was 1.0 days (p25 = 0.40, p75 = 2). Postoperative outcomes showed a corticosteroid reduction in 52 participants (61%), and 30 (35%) showed anticholinesterase reduction. The median dose of corticosteroids before surgery was 40.00 mg, and after surgery, 20.00 (*p*-value < 0.001). Based on Osserman classification, before surgery, it was observed that 4% had grade I, 15% grade IIa, 32% grade IIb, 31% grade III, and 18% grade IV. After surgery, it was observed that 28.5% were asymptomatic, 28% had grade I, 34% had grade IIa, 3.6% had grade IIb, and 5.9% had grade III. There was no statistical difference in clinical and pharmacological response in the analysis with and without thymoma and myasthenia (*p*-value 0.403; *p*-value 0.104). About the surgical approach, patients undergoing thymectomy by sternotomy have longer hospital and ICU stays with statistical significance (*p*-value <0.001; p-value 0.005).

**Conclusion:**

This study demonstrated that surgical treatment for myasthenia gravis is safe and effective for symptom control and medication reduction, regardless of the surgical approach, with shorter ICU and hospital stays through the minimally invasive approach.

## Introduction

Myasthenia gravis (MG) was first described in the medical literature by the English physician Thomas Willis in 1672. The term myasthenia gravis originates from the Greek word “myasthenia,” meaning muscle weakness, while the term “gravis” is derived from Latin, meaning severe. This term was first used by Friedrich Jolly in 1895 (1).

MG is a rare, chronic, autoimmune disease that affects the neuromuscular junction. Despite its rarity, it is the most common disease of the neuromuscular junction. The incidence of the disease ranges from 4.1 to 30 cases per million person-years, and the prevalence rate varies from 150 to 200 cases per million (2, 3).

The pathophysiology of myasthenia is complex and not fully understood. MG is largely a treatable disease, but it can result in significant morbidity and even mortality. This is typically preventable with timely diagnosis and appropriate treatment (1, 3).

Acetylcholine receptor antibodies are seen in most MG patients, approximately 90%. However, other antibodies have been discovered over the past 20 years, including muscle-specific kinase (MuSK) and low-density lipoprotein receptor-related protein 4 (LRP4), and are now available through commercial tests. Additionally, there exists another group known as “seronegative,” where none of these antibodies are identified (3, 4).

It is important to note that thymectomy has a proven effect and is indicated for cases with acetylcholine receptor antibodies. In anti-MuSK cases, its ineffectiveness has been demonstrated, and thus, surgery should not be indicated. For the seronegative group, the role of surgery remains inconclusive (4).

The main clinical manifestation of MG is fatigable muscle weakness, worsened by exertion and improved by rest. The most common symptoms are ocular, with double vision and ptosis. Most patients will develop diplopia and/or ptosis at some point during the disease course. Furthermore, up to 80% of patients with ocular onset will develop generalized symptoms, usually within two years of disease onset (1, 5).

The treatment of myasthenia gravis involves the use of anticholinesterase agents, immunomodulators (biological immunotherapies and corticosteroids), and surgical treatment via thymectomy (6, 7).

The first description of thymectomy for the treatment of MG was made 75 years ago by Blalock, in 6 patients, who underwent surgery, with 3 patients showing favorable outcomes (8, 9).

Different types of surgical techniques for thymectomy have been described, mainly differing in the surgical access used. Among the various approaches described are: median sternotomy, partial sternotomy (manubriotomy), lateral thoracotomy, cervicotomy, median sternotomy combined with cervical incision, median sternotomy combined with subxiphoid incision, videothoracoscopy in unilateral and bilateral modalities; more recently, robotic thymectomy has also been described. Regarding the extent of resection, thymectomies can be performed by removing only the thymic gland or by removing the gland along with the peri-thymic adipose tissue, from the upper poles of the thymus in the cervical region to the diaphragm base in a block resection with direct visualization, preserving the phrenic and vagus nerves (6, 10).

Despite the impact of this study on global clinical practice, many questions remain unanswered, such as: which is the best access route, the current complication rates varying according to the access route, and what are the predictors of success, among others. The Thoracic Surgery Service at the Clinics Hospital FMUSP, in collaboration with the Neurology Department of FMUSP, in line with the literature trend, began performing videothoracoscopic thymectomies for MG patients requiring surgical treatment in 2005. This accumulated experience may help address some of these unanswered questions.

The present study was developed to assess the outcomes in patients undergoing thymectomy, focusing on the effectiveness and safety of thymectomy for the treatment of myasthenia gravis.

## Methods

Data were collected from the database of the Department of Thoracic Surgery at the Clinics Hospital of the University of São Paulo. All medical records of patients who underwent thymectomy for the treatment of myasthenia gravis between 2014 and 2024 were extracted from the database. A total of 103 medical records were initially retrieved. Patients with incomplete data, myasthenia gravis subtypes with negative AChR antibodies, or those without postoperative follow-up in the Neurology and Thoracic Surgery outpatient clinics were excluded from this study.

The data were presented using descriptive statistics, including mean, median, standard deviation, and 1st and 3rd quartiles (p25 and p75) for quantitative variables, and absolute numbers and relative percentages for categorical variables. To investigate the effect of surgery on the database variables, the Kruskal-Wallis ANOVA was used, with Dunn’s *Post Hoc* test when necessary. To investigate the association between surgical success, evaluated by a reduction in the use of anticholinesterase drugs and/or a reduction in corticosteroid use, logistic regression models were constructed. A simple model was built to obtain the Odds Ratio with its respective 95% confidence intervals for the unadjusted model, and an adjusted model was created, accounting for the participants’ age, to obtain the Odds Ratio with its 95% confidence intervals for the adjusted model. To compare the variables between groups that reduced or did not reduce the use of anticholinesterases and corticosteroids, the Mann–Whitney test was used for quantitative variables and the Chi-square test or Fisher’s Exact test for categorical variables. All analyses were performed in the R programming environment, adopting a significant level of 5% (*p* < 0.05).

Medication data were extracted from the visit closest to three months before surgery and the one closest to 12 months after surgery. Pre- and postoperative Osserman classification was used to assess clinical status. Thymic volume, in grams, was recorded from pathology reports.

## Results

The sample of this study consisted of 85 participants, with ages ranging from 13 to 72 years (mean = 43, SD = 14 years). Most of the participants were female (75%), and the median time from diagnosis was 3 years (p25 = 1; p75 = 5). Regarding the type of surgery, 21 patients (approximately 25%) underwent open surgery via sternotomy, while 64 patients (approximately 75%) underwent minimally invasive surgery via videothoracoscopy. The median total hospital stay for the participants was 4.0 days (p25 = 3.0; p75 = 5.5), with the median time in the general ward being 2.2 days (p25 = 2.0; p75 = 4.0) and the median stay in the ICU being 1.0 day (p25 = 0.40; p75 = 2). All patients undergoing thymectomy tested positive for anti-AChR antibodies. Table 1 below presents the general characteristics of the participants according to the type of surgery performed.

**Table 1:** General characteristics of the population.

Variable	Total (*N* = 85)	Sternotomy (*N* = 21)	VATS (*N* = 64)	*p*-value
Age, years (mean ± SD)	43 (±14)	49 (±11)	38 (±12)	0.009
Sex, *n* (%)				0.357
Female	64 (75%)	14 (67%)	50 (78%)	
Male	21 (25%)	7 (33%)	14 (22%)	
Disease duration, years (median, IQR)	3.0 (1.0; 5.0)	3.0 (1.0; 5.0)	3.0 (1.0; 5.5)	0.689
Length of stay (days, median, IQR)				
Ward	2.2 (2.0; 4.0)	2.5 (2.0; 4.0)	2.0 (1.0; 3.0)	0.121
ICU	1.0 (0.4; 2.0)	2.0 (1.0; 2.0)	1.0 (0.9; 2.0)	0.005
Total	4.0 (3.0; 5.5)	4.0 (3.0;6.00)	4.0 (3.5–6.0)	<0.001

Mean (± SD); *n* (%); Median (p25, p75).

Kruskal-Wallis ANOVA; Fisher’s exact test.

Regarding the characteristics of the population undergoing thymectomy for the treatment of myasthenia gravis, patients who underwent minimally invasive surgery had a shorter hospital stay, with statistical significance (4.0 vs. 3.0, *p* < 0.001).

Analyses regarding the hospital stay in the ICU and in the general ward were performed, with a longer stay in the ICU for patients undergoing conventional thymectomy, showing statistical significance (2.0 vs. 1.0, *p* = 0.005). No statistically significant differences were found regarding the length of stay in the general hospital ward (Table 2).

**Table 2:** Pre and postoperative medication doses.

Variable	Preoperative	Postoperative	*p*-value
Corticosteroid doses	40.0 (20.0; 40.0)	20.0 (10.0; 40.0)	< 0.001
Anticholinestese doses	240.0 (240.0; 360.0)	240.0 (240.0; 300.0)	0.30

Mean (± SD); *n* (%); Median (p25, p75).

**Figure 1:**
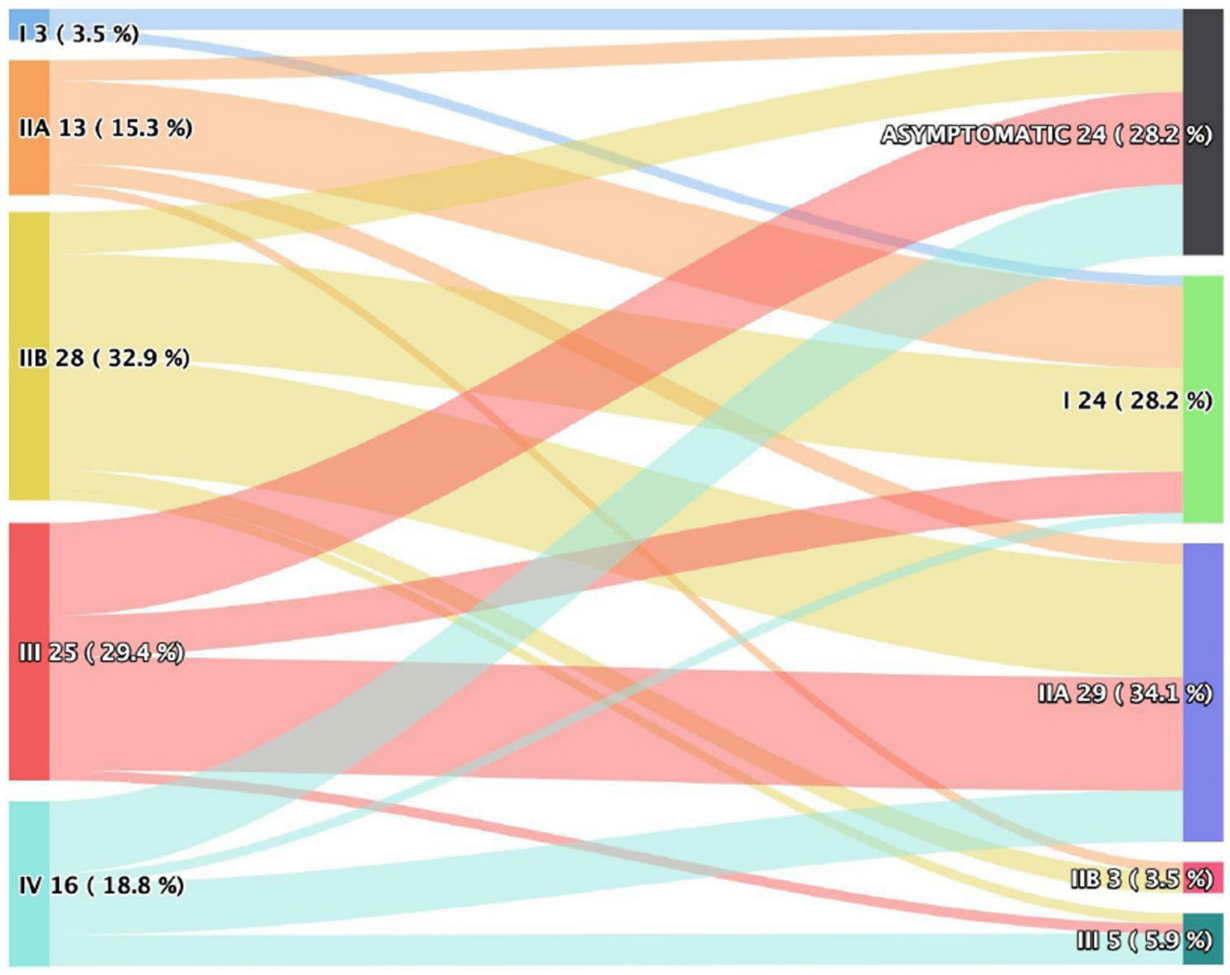
Osserman classification pre and postoperative.

Patients were classified according to the clinical manifestations of myasthenia gravis based on the Osserman scale. The patients included in this study were followed by the Thoracic Surgery and Neurology teams during the preoperative period and up to 12 months postoperatively. They were categorized into groups before and after surgery. Regarding the preoperative Osserman classification, 3 patients (3.5%) were grade I, 13 (15.3%) were grade IIa, 28 (32.9%) were grade IIb, 25 (29.4%) were grade III, and 16 (18.8%) were grade IV. After one year following the surgery, it was observed that 24 patients (28.2%) were asymptomatic, 24 (28.2%) were grade I, 29 (34.1%) were grade IIa, 3 (3.5%) were grade IIb, and 5 (5.9%) were grade III. No patients were classified as grade IV (myasthenic crisis) after surgery. Figure 1 shows the comparison of Osserman classification pre- and postoperatively. Unfortunately, subtyping (A/B) was not systematically recorded in our dataset for grades III and IV.

The impact of surgery on the treatment of myasthenia gravis is reflected in the reduction of corticosteroid dosage, with statistical significance. The median corticosteroid dose before surgery was 40.0 mg (20.0–40.0 mg), and after surgery, it was 20.0 mg (10.0–40.0 mg), with a *p*-value < 0.001. The dose of anticholinesterase medications showed no statistical significance (240.0 vs. 240.0, *p* = 0.3). Table 2 shows the comparasion of pre and postoperative medication doses.

Analyses regarding the volume of thymic tissue removed and the reduction in anticholinesterase and corticosteroid doses were conducted. Patients with a reduction in medication doses had a larger volume of thymic tissue removed compared to patients who did not reduce medication doses, though this difference was not statistically significant (118.0 g vs. 86.0 g, *p* = 0.3).

Bivariate analysis between disease duration and clinical improvement was demonstrated that patients with shorter disease duration were significantly more likely to achieve clinical improvement after thymectomy (*p* < 0.001; Mann–Whitney U test).

Another bivariate analysis between pre-treatment Osserman classification and clinical improvement was demonstrated that patients in lower Osserman classes (I, IIA, IIB) had significantly higher odds of clinical improvement compared to those in higher severity classes (III–IV) (*p*-value 0.004; chi-squared test).

A logistic regression model was developed to evaluate the combined effect of disease duration and Osserman classification (pre-treatment) on clinical improvement (Table 3).

**Table 3:** Myasthenia and thymoma population characteristics.

	Thymoma		
Variable	No (*N* = 64)	Yes (*N* = 21)	*p*-value
Anticholinesterase reduction			0.403
No	43 (67%)	12 (57%)	
Yes	21 (33%)	9 (43%)	
Corticosteroid reduction			0.104
No	28 (44%)	5 (24%)	
Yes	36 (56%)	16 (76%)	
Class reduction			0.191
No	8 (13%)	0 (0%)	
Yes	56 (88%)	21 (100%)	
ICU stay, days	1.00 (1.00; 2.00)	1.00 (0.00, 1.50)	0.138
Hospital stay, days	4.00 (3.00; 6.00)	4.00 (3.00, 5.00)	0.903

*N* (%); Median (Q1, Q3); Pearson’s Chi-squared test; Fisher’s exact test; Mann–Whitney (Wilcoxon rank sum test).

For every additional year of disease duration, the odds of clinical improvement decreased by 12% [Odds Ratio (OR) = 0.88, *p* < 0.001] and patients classified in higher severity classes (III–IV) had significantly reduced odds of clinical improvement compared to those in lower severity classes (I–IIA) (*p* < 0.05).

Thymoma and myasthenia gravis overlap are an even rarer condition than myasthenia gravis alone. Analyses were performed on the overlap of thymoma and myasthenia gravis and its impact on medication dose reduction. In the present sample, 21 patients presented this overlap, being, according to the WHO classification, three patients A; five patients AB; six patients B; five patients B2 and two patients B3. The reduction in anticholinesterase dose was observed in only 9 patients, while 16 patients had a reduction in corticosteroid dosage. However, in both groups, there was no statistical significance (*p* = 0.403 vs. 0.104). All patients with the overlap of conditions showed a reduction in their medication category. Analyses regarding the ICU stay and total hospital stay were performed, with no statistical significance (*p* = 0.138; *p* = 0.903). Table 3 demonstrates the population with overlapping thymoma and myasthenia gravis.

Postoperative complication analyses were performed. Diaphragmatic paralysis occurred in nine patients (11%), diagnosed by X-ray, all unilateral, with spontaneous resolution within 3 months without need for intervention. Other complications were also analyzed, including wound infection in three patients. Four patients (4.7%) experienced non-crisis exacerbations (e.g., transient ptosis, dysphagia) and were managed conservatively as outpatients.

## Discussion

Thymic tissue resection as an adjunctive treatment in patients with myasthenia gravis is a well-studied and well-established condition in the medical literature. The present study aimed to evaluate the characteristics and clinical outcomes of patients undergoing thymectomy for the treatment of myasthenia gravis, focusing on the differences between surgical approaches (open vs. minimally invasive), as well as the clinical evolution of patients over time (4, 8, 11).

The sample consisted of 85 participants, with a mean age of 43 years, and a predominance of females, which is consistent with the literature indicating a higher prevalence of myasthenia gravis in females (1, 2). The age range in the sample, from 13 to 72 years, reflects the heterogeneity of the patient profile affected by the disease, which can affect individuals from various age groups.

Different techniques for thymectomy have been described and refined over the years. The development of the minimally invasive approach has gained prominence in the surgical treatment of myasthenia gravis. The comparison between the types of surgical approaches showed that patients who underwent the minimally invasive approach had a shorter hospital stay, with a significant difference (4.0 vs. 3.0, *p* < 0.001), which corroborates findings from other studies that highlight faster recovery associated with less invasive techniques (8, 12–14). This finding was also reflected in the ICU stay analysis, where patients in the conventional approach had a longer stay (2.0 vs. 1.0, *p* = 0.005), possibly due to the greater impact of sternotomy on postoperative recovery, as previously discussed in another research (4, 15). On the other hand, there were no significant differences in the general ward stay, suggesting that both surgical groups may have a similar recovery in this regard.

The clinical presentation of patients with myasthenia gravis is broad, ranging from ocular manifestations to those with muscular involvement. There are clinical and functional classifications for myasthenia gravis. Regarding the Osserman clinical classification, a substantial improvement in the clinical status of patients was observed after thymectomy, with 28.2% of participants becoming asymptomatic and 28.2% reaching grade I on the Osserman scale at one year of follow-up. This data supports the results of previous studies indicating that thymectomy has a significant positive impact on the clinical course of myasthenia gravis, leading to a reduction in the clinical manifestations of the disease (11, 14).

Thymic tissue resection, in addition to having a positive impact on clinical manifestations, also shows a positive impact on medications that are part of the therapeutic arsenal for myasthenia gravis. The present study revealed a significant reduction in corticosteroid dosage post-surgery, with a median of 40 mg before the procedure and 20 mg after surgery (*p* < 0.001). This reduction in corticosteroid use is consistent with the literature, which suggests that thymectomy can decrease the need for immunosuppressants, contributing to more effective disease control with a lower risk of long-term side effects (8, 16). However, the dose of anticholinesterase medications did not show significant change, which may indicate that, although thymectomy favors a reduction in corticosteroid use, disease control with anticholinesterase medications may still be necessary for many patients.

Although patients with a larger volume of thymic tissue resected showed a trend toward reduced medication doses, there was no statistically significant difference between groups, suggesting that other factors beyond tissue volume may be influencing the response to medication reduction. Similar studies indicate that the volume of tissue resected may not be the sole determinant for the clinical response to surgery (3, 13).

About the association between disease duration and clinical improvement in our study, patients was submitted for a thymectomy with shorter disease duration were significantly more likely to achieve clinical improvement after thymectomy, as we saw in MGTX study (4).

The overlap of myasthenia gravis and thymic tumors (thymomas) is described in the literature as occurring in approximately 10–15% of cases (2, 5). In the present study, 24% of patients had this overlap. The analysis of patients in this group did not show a significant difference in medication dose reduction, even though all patients with thymomas experienced a reduction in their therapeutic class. This is important because it suggests that the presence of thymomas does not prevent medication reduction in patients with myasthenia gravis, but the lack of statistical significance regarding the doses may indicate greater complexity in these cases, requiring further studies to understand the exact impact of thymic tumors on disease treatment (9).

Regarding postoperative complications, transient diaphragm paralysis was the most common (11% of cases), with spontaneous resolution, which is a recognized complication in thymectomy studies (13). No myasthenic crises occurred in the immediate postoperative period, which is a positive outcome, as severe complications of this nature are often associated with less favorable clinical outcomes.

## Limitations and final considerations

Although this study provided important insights into the impact of thymectomy in the treatment of myasthenia gravis, several limitations should be considered. The sample of 85 participants, while reasonable, could be expanded to include a greater diversity of patients, especially in terms of age, comorbidities, and other clinical characteristics that may influence postoperative outcomes. The study is limited by its retrospective, single-center design, lack of a non-surgical control group, and absence of standardized scales such as MGFA-PIS or QMG. Subtype data (e.g., Osserman IIIA/IIIB) were not available.

In conclusion, the results of this study indicate that thymectomy, especially via minimally invasive approach, can be an effective option in the treatment of myasthenia gravis, leading to significant clinical improvement and reduction in corticosteroid use. However, further studies are needed to deepen the understanding of the factors influencing the surgical response, particularly in cases with thymoma overlap or in patients with more severe forms of the disease.

Future studies should explore robotic thymectomy, long-term remission rates, and prospective comparisons including standardized MG severity scales.

## Data Availability

The original contributions presented in the study are included in the article/supplementary material, further inquiries can be directed to the corresponding author.
